# Peer feedback for examiner quality assurance on MRCGP International South Asia: a mixed methods study

**DOI:** 10.1186/s12909-017-1090-1

**Published:** 2017-12-08

**Authors:** D. P. Perera, Marie Andrades, Val Wass

**Affiliations:** 1 Department of Family Medicine, Faculty of Medicine, University of Kelaniya, Ragama, Sri Lanka; 2 Department of Family Medicine, Aga Khan University Hospital, Karachi, Pakistan; 3 Emeritus Professor of Medical Education, Faculty of Health, Keele University, Newcastle under Lyme, UK

**Keywords:** Examiner quality assurance, Peer feedback, MRCGP [INT] examination

## Abstract

**Background:**

The International Membership Examination (MRCGP[INT]) of the Royal College of General Practitioners UK is a unique collaboration between four South Asian countries with diverse cultures, epidemiology, clinical facilities and resources. In this setting good quality assurance is imperative to achieve acceptable standards of inter rater reliability. This study aims to explore the process of peer feedback for examiner quality assurance with regard to factors affecting the implementation and acceptance of the method.

**Methods:**

A sequential mixed methods approach was used based on focus group discussions with examiners (*n* = 12) and clinical examination convenors who acted as peer reviewers (*n* = 4). A questionnaire based on emerging themes and literature review was then completed by 20 examiners at the subsequent OSCE exam. Qualitative data were analysed using an iterative reflexive process. Quantitative data were integrated by interpretive analysis looking for convergence, complementarity or dissonance. The qualitative data helped understand the issues and informed the process of developing the questionnaire. The quantitative data allowed for further refining of issues, wider sampling of examiners and giving voice to different perspectives.

**Results:**

Examiners stated specifically that peer feedback gave an opportunity for discussion, standardisation of judgments and improved discriminatory abilities. Interpersonal dynamics, hierarchy and perception of validity of feedback were major factors influencing acceptance of feedback. Examiners desired increased transparency, accountability and the opportunity for equal partnership within the process. The process was stressful for examiners and reviewers; however acceptance increased with increasing exposure to receiving feedback. The process could be refined to improve acceptability through scrupulous attention to training and selection of those giving feedback to improve the perceived validity of feedback and improved reviewer feedback skills to enable better interpersonal dynamics and a more equitable feedback process. It is important to highlight the role of quality assurance and peer feedback as a tool for continuous improvement and maintenance of standards to examiners during training.

**Conclusion:**

Examiner quality assurance using peer feedback was generally a successful and accepted process. The findings highlight areas for improvement and guide the path towards a model of feedback that is responsive to examiner views and cultural sensibilities.

**Electronic supplementary material:**

The online version of this article (10.1186/s12909-017-1090-1) contains supplementary material, which is available to authorized users.

## Background

The International Membership Examination (MRCGP[INT]) of the Royal College of General Practitioners UK (RCGP UK) was developed by leaders in Family Medicine from Bangladesh, India, Nepal, Pakistan and Sri Lanka. Expertise of family physicians from all member countries is pooled in the design of the assessment process. This ensures that the exam is appropriate for family doctors from all member countries as differences in culture, epidemiology, clinical facilities and resources are considered. An RCGP UK appointed Internal Development Advisor, supports the curriculum review process and development and implementation of assessments. RCGP External Development Assessors, quality assure and accredit exams at fixed intervals [1]. MRCGP [INT] SA was originally accredited in 2007 and re-accredited in 2010 and 2013.

The examination conducted in English consists of a 200 item single best answer applied knowledge test paper and an objective structured clinical examination (OSCE) consisting of 14 ten minute stations. The OSCE exam is conducted alternately in Pakistan and Sri Lanka. Examiners for the OSCE are mainly from Bangladesh, India, Pakistan and Sri Lanka.

The MRCGP[INT] SA examination presents a challenge in that it is a collaboration between four countries. Throughout the development of the exam careful consideration of the background differences have been made to achieve an assessment of high standard and compatibility across countries. For example due to inability to achieve consensus on the definition of professionalism which is a culturally sensitive social construct; modifications were made to the original marking scheme [2, 3].

Examiners on MRCGP[INT] SA face a rigorous selection and training process as well as strict quality assurance procedures. Examiners must have the relevant academic qualifications and participate in a group activity session that tests their team skills followed by an interview. Wakeford et al. stated that examiners need knowledge and skills relating to their subject as well as the design and conduct of the examination [4]. In keeping with this concept it is mandatory for examiners on the MRCGP[INT] OSCE to be practising family physicians. All examiners participate in OSCE case writing and case piloting. During the duration of each OSCE examination there are several video marking sessions of candidate performances each morning. These sessions are followed by group discussions that aim to help harmonise examiner standards. Examiners also routinely receive feedback from a psychometric consultant on their performance at the end of every OSCE examination. They are provided information regarding their mean pass rates, inter rater reliability, and distribution of grades.

Many researchers have reported that there is no evidence that training improves examiner performance [5]. Others have reported that examiner training and monitoring are integral to improving and maintaining exam standards [6, 7]. Wakeford et al. in writing on the MRCGP UK oral examinations stated that unless examiners are carefully selected, trained, and monitored, examinations may become haphazard [4]. Khera et al. described willingness to accept training and regular monitoring of performance as one of the desirable attributes of a clinical examiner [6]. The Australasian College of Emergency Medicine has instituted an examiner peer review process with a small pool of senior examiners, who observe examiner candidate interactions and complete assessment forms on examiner techniques. This process has become accepted by examiners as constructive feedback and an essential element of peer support [8].

A peer feedback process takes place during the exam as one of the main methods of examiner quality assurance. It is an integral aspect of quality assurance of the exam through monitoring examiner performance and maintaining and enhancing examiner skills. Peer reviewers are members of the OSCE core group who organise the OSCE examination. They have experience as examiners on the MRCGP[INT] SA OSCE and regularly participate as examiners on the OSCE themselves. During the course of each OSCE exam members of the OSCE core group sit in with each examiner. They observe examiner competency in global assessment, consistency and reliability and double mark over a few candidates. During breaks in between candidates, reviewers provide verbal feedback to the examiner which is documented and placed on record.

Literature related to the training of clinical examiners in the Asian context is limited. Peer feedback in various settings has revealed conflicting evidence of usefulness, or acceptance [9]. We do not know how well this process is accepted given the complexities of the South Asian exam.

This study investigated the quality assurance process of peer feedback with regard to factors affecting the implementation and acceptance of the method in this cross cultural setting with a view to contributing to continuous development and improvement of the overall assessment.

## Methods

A sequential exploratory mixed methodology was used to gain an indepth view of this subject which is a little researched area [10]. The qualitative data helped develop an understanding of the relevant issues. The anonymous questionnaire enabled objective quantification of the categorised issues, refining of the issues and gathering of information from a wider group. It also helped with “giving voice to different perspectives” facilitating candid disclosure of opinions balancing out the focus group discussion (FGD) findings that could be weighted by more dominant voices [11].

The preliminary stage was conducted in March 2012 during an OSCE exam for 113 candidates in Pakistan. Two focus group discussions attended by six examiners (FGDE) each were conducted with eight male and four female examiners out of the 23 examiners present throughout the duration of the exam (Sri Lanka 2, Pakistan 19, Bangladesh 1, United Kingdom 1) by the principal investigator (DP) (Table 1). The initial topic guide based on the relevant literature explored the utility of peer feedback, reactions to feedback, factors affecting the response to feedback, and format of giving feedback. Each focus group lasted approximately 90 min and was recorded and transcribed. A focus group discussion was also conducted with all four peer reviewers (FGDR) who were female: three from Pakistan and one from Sri Lanka and in the age range 37 to 45 years. All reviewers had been examiners on the MRCGP[INT] OSCE more than three times with previous experience in giving feedback and had received some training in giving feedback.

**Table 1:** Examiner focus group participants (FGDE)

Country	Gender	
	Male (M)	Female (F)
Bangladesh (BD)	2	…
India (I)	…	…
Pakistan (Pak)	4	4
Sri Lanka (SL)	2	…

A Questionnaire (Additional file 1) was formulated for examiners by summarising the themes emerging from the qualitative component of the study to enable coding as yes or no answers; a method known as “quantitisation of qualitative data” [12]. The questionnaire was piloted on one examiner. Quantitative data was gathered from 14 male and 6 female examiners aged 36 to 64 years (response rate 77%) using the anonymous self-administered questionnaire following open invitation to all 26 examiners (SL 12, Pak 12, BD 1, UK 1) present at the September 2012 OSCE exam in Sri Lanka taken by 155 candidates some of whom had participated in the preliminary FGDs (Tables 2, 3 and 4).

**Table 2:** Questionnaire participants (Q)

Country	Gender	
	Male (M)	Female (F)
Bangladesh (BD)	2	…
India (I)	…	…
Pakistan (Pak)	7	5
Sri Lanka (SL)	5	1

**Table 3:** Participant responses to questionnaire regarding utility, interpersonal dynamics, equity, hierarchy and validity of feedback (*n* = 20)

	Yes, *n* (%)	No, *n* (%)
Would you prefer a remote form of observation to direct observation? eg. By video recording candidate and examiner performance	13 (65)	7 (35)
Has your acceptance of feedback varied depending on who is giving you feedback? Eg. Senior member of the team/junior member of the team	11 (55)	9 (45)
Has your acceptance of feedback varied depending on the way it was delivered?	11 (55)	9 (45)
Has your level of comfort with the peer feedback process improved with increased number of times you experienced review?	20 (100)	–
Do you find you have adequate opportunity for discussion with your reviewer?	7 (35)	13 (65)
Did the reviewers’ comments/presence help you in your marking in any way?	16 (80)	4 (20)
Would you like to have a written copy of the reviewers’ feedback?	18 (90)	2 (10)
Would you like more detailed feedback?	13 (65)	7 (35)

**Table 4:** Participants views regarding reviewers (n = 20)

	Agree *n* (%)	Disagree *n* (%)	Not sure *n* (%)
Reviewers should be members of the OSCE group	14 (70)	6 (30)	–
Reviewers should be selected from common pool of examiners	16 (80)	3 (15)	1 (5)
All reviewers should undergo regular QA through peer feedback	14 (70)	6 (30)	–
In pairing examiner to reviewer matching of seniority should be attempted as far as possible	13 (65)	4 (20)	3 (15)
Reviewers should be senior members of the team only	6 (30)	12 (60)	2 (10)
Junior examiners on the team can be efficient reviewers too	13 (65)	1 (5)	6 (30)
Regular external reviewer participation is important eg. RCGP representatives not directly involved with exam process	17 (85)	1 (5)	2 (10)

Qualitative data from the FGDs and free text questions from the questionnaire were analysed through a process of reflexive iteration of the initial and emerging themes. Initial coding was done by the principal investigator (DP) and repeatedly revisited and revised using a constant comparative method. Findings were shared and discussed with the other investigators (MA, VW) through e-mail and face to face discussion. The main themes that emerged are given below.

The quantitative data were analysed separately and in integration of qualitative and quantitative data; quantitative data were scrutinised for corroboration with the qualitative findings (convergence), complementarity of findings to facilitate deeper insights or contradiction between quantitative and qualitative findings (dissonance) [12].

## Results

### Qualitative findings

#### The utility of peer feedback

There was general consensus among examiners that peer feedback was an important aspect of quality assurance. “*It’s a helpful tool.*” (SL, M, FGDE, >3) “*Helpful in making me more confident.”* (Pak, F, Q, 1) Examiners realised that self appraisal was inadequate for improvement and maintenance of standards. “*Sometimes we cannot find out our own shortcomings.”* (BD, M, FGDE, >3).

Examiners gave specific examples of how peer feedback contributed to improvement of their skills*. “Helped to spread out marks and discriminate between candidates.”* (SL, M, Q, 2).

“*The discussion helped me to shift my thinking to very objective.”* (Pak, M, Q, 1).

Many examiners requested more detailed feedback.

### Interpersonal dynamics of implementing the peer feedback process

Some difficult experiences and conflicting ideas related to interpersonal dynamics during the feedback process were discussed.

Interpersonal dynamics had a major impact on level of comfort with giving and receiving feedback. Reviewers and examiners emphasised that their level of comfort was dependent on the specific individual they were dealing with. One examiner stated “*It depends on who is sitting with you”* (Pak, F, FGDE, > 3) and reviewers echoed this sentiment saying, *“It depends on who you are giving feedback to”* (FGDR) and “*How I think it will be perceived.” (FGDR)*.

Reviewers acknowledged the challenges of negotiating the feedback process. “*We are also learning how to give feedback.”* (FGDR).

The manner in which feedback was given appeared to be strongly related to the acceptance of feedback.

“*Feedback given in a nonjudgmental blame free environment is easier to accept.”* (BD, M, Q, >3).

“*One reviewer was very dogmatic with her opinions and sounded very critical – difficult to accept but still very helpful.”* (SL, M, FGDE, 2).

The process was a stressful experience for the examiners and reviewers.

*“Remote observation would have been better.” *(BD, M, FGDE, >3).

*“Somebody sitting by your side when you are marking makes you a little anxious.” *(SL, M, FGDE, 2).

Reviewers felt that examiner behaviour changed in their presence. Examiners were stated to become more “*alert” (FGDR), “uncomfortable”* (FGDR), or “*they become more defensive; you can see it in their body language.” (FGDR).*

Examiners stated that anxiety decreased with increased number of times they had been quality assured.

Reviewers reported that it was uncomfortable providing negative compared to positive feedback. Reasons given for discomfort with providing critical feedback were fear of the reaction to feedback, specific personality of the person receiving feedback and hierarchical factors.

There was evidence of resistance to accepting negative feedback as well.

“*Somebody who is judging you may have different ideas different standards.”* (SL, M, FGDE, 2).

Some examiners revealed fears of the repercussions of receiving negative feedback. “*Helpful to know that it’s not going to be used against you.”* (SL, M, FGDE, 2).

Examiners wanted a clearer idea on what the feedback given to them meant.

“Sometimes it is not understandable.” (BD, M, FGDE, >3).

“Must make it more simple or must explain what it means.” (Pak, F, FGDE, 2).

### Transparency, accountability and a desire for equal partnership within the feedback process

Examiners demonstrated a desire to want to be engaged partners in the process in opposition to passive acceptance of feedback. The following comments illustrate these views.

Examiners wanted a written copy of the peer feedback they received. Reasons given were; for future reference and further reflection and a possible desire to increase transparency and accountability of reviewers.

“*Giving it in black and white their judgment will be more objective.” (*BD, M, FGDE, >3).

Examiners wanted to be able to give written responses to the feedback given to them. Reviewers welcomed this idea. “*I think it is a very positive thing to have a response.*” (FGDR).

Many examiners wanted to see selection of some of the reviewers from the general pool of examiners. “*I think group of people including senior members from different countries should give feedback.”* (Pak, M, FGDE, 1).

Some examiners stated that having two different reviewers on two different days would provide a more “*balanced*” quality assurance report.

Examiners stated that quality assurance should periodically be done by external reviewers not connected to the organising team such as RCGP representatives who conduct accreditation of the exam. However some examiners expressed greater anxiety associated with external review. “*I was more uncomfortable with the external reviewers doing accreditation than our reviewers.”* (Pak, F, FGDE, 1).

### The impact of hierarchy

There was an emphasis on the impact of hierarchy in the acceptance of feedback. Senior and junior examiners alike were more likely to accept feedback given by a person senior to them.

*“Feedback given by more experienced persons is more valuable.” (Pak, M, Q, 3).*

*“I respect the feedback from a senior person.” (BD, M, Q, >3).*

*“Senior members are better to guide.” (*Pak, M, FGDE, 1).

Examiners suggested that matching examiner to reviewer seniority as far as possible would be better practice.

However a comment made by one examiner underlines the highly individual nature of hierarchical perceptions.

“*I was given feedback by three people all younger in age but richer in experience. That didn’t bother me.”* (Pak, M, Q, 2).

Some reviewers revealed difficulty giving feedback to a senior examiner who was more experienced than themselves.

*“I’m not 100% comfortable”* when giving feedback to a senior examiner. (FGDR).

“*Junior examiners are more receptive and they expect that they might get negative feedback.” (FGDR).*

### Examiner perceptions regarding the validity of feedback

Examiners had much to say on the process of standardisation and selection of reviewers. They emphasised the importance of rigorous procedures for the selection and standardisation of reviewers to ensure feedback was valid. There were concerns about impartiality and objectivity.

“*Reviewers should be calibrated, standardised it should not be their personal opinions.”* (BD, M, FGDE, >3).

*“Three to four examiners should QA reviewers before they are selected as reviewers and with the correlation scrutinised.”* (P, M, FGDE, >3).

Examiners stated that reviewers should participate as examiners on the exam regularly and that reviewers should regularly be quality assured through peer feedback.

Examiners and reviewers showed interest in participating in training workshops. Reviewers suggested that workshops on feedback methodologies should be conducted for examiners as well as reviewers to highlight the importance of the peer feedback process for quality assurance as well as educate on the need to be receptive to constructive criticism. Specific areas that training was requested in were the approach to giving feedback to people with difficult personalities, colleagues who were senior and more experienced or were personal friends. “*General training on method of giving constructive criticism in a way that would be accepted and bring about an improvement in performance.”* (FGDR). Reviewers stated that a structured feedback format might improve the objectivity of their feedback.

### Quantitative findings

The quantitative data from the questionnaire helped to expand on many of the qualitative findings. The data strongly corroborated and strengthened the qualitative findings regarding; increase in comfort with increased number of times receiving feedback, perception of usefulness, request for written feedback and the desire for regular external reviewer participation in examiner QA.

Dissonance between qualitative and quantitative findings could be seen in two areas. Regarding recruitment of reviewers, 80% thought that reviewers should be selected from the common pool of examiners, however 70% thought that reviewers should be members of the OSCE group. While the FGDs revealed an overarching influence of hierarchy on acceptance, 60% rejected the suggestion that only senior members could be efficient reviewers and 65% accepted that junior members of the team could be efficient reviewers.

## Discussion

Peer feedback aims to facilitate improvement and behaviour change similar to formative assessment which guides future learning, provides reassurance, promotes reflection and shapes values [13]. Epstein et al. stated that limitations in the ability of humans to know themselves as others see them restrict the usefulness of self-assessment. Examiners in this study showed awareness of the limitations of self assessment [13]. They specifically stated that peer feedback gave them an opportunity for discussion, standardisation of judgments and improved discriminatory abilities through spread of gradings for the components tested.

It is reported that although peer assessment has the potential to provide accurate and valid assessment information, several factors such as reliability, relationships and stakes influence the quality of the results [14]. Examiners in our study had similar concerns. They discussed the impact of prior relationships, hierarchy and possible repercussions of poor feedback. These findings relate to the fact that feedback in any form is never delivered or received in a vacuum. Any feedback that is delivered will be interpreted through the filters with which the receiver views the world of practice, the feedback provider, and his abilities [15]. Inappropriately delivered feedback can be ‘undermining, destructive, and divisive’ [13]. Participants in this study stressed that acceptance had largely been dependent on how the feedback was delivered and requested more opportunities for discussion with their reviewer. Similar findings were seen in a study of acceptance of feedback by academics in a clinical teaching setting [16]. It is important that examiners view the peer feedback process as an opportunity to actively engage in the continuous improvement and maintenance of examination standards. Better dialogue could lead to feedback being perceived as a tool for their improvement rather than as a destabilising or debilitating act ‘done to them’ by those in authority [17]. Participants in this study suggested that more formal documentation procedures could help make the process more transparent and increase the accountability of participants.

Studies on peer feedback in other settings have emphasised that peer feedback should take place in an atmosphere of trust and confidentiality [18]. The major body of literature on feedback advocates that feedback should be timely in order to be most effective however the situation may be more complex [14, 19]. In this study feedback was given to examiners within the stressful environment of an examination making the feedback instantaneous but not necessarily in an ideal safe environment. The environment and the emotional state of the person receiving feedback should therefore be taken into account when determining the optimal time for feedback encounters [17].

The likelihood of acceptance of any feedback given will depend on the perceived validity of feedback. The credibility of the source of feedback and the rigor of the selection process of reviewers was a main determining factor for acceptance of feedback by examiners. This was also seen in a study on multisource feedback where physician reactions to peer feedback were influenced by perceptions of accuracy, credibility and usefulness of feedback. Factors shaping these perceptions included: recruiting credible reviewers, ability of reviewers to make objective assessments, use of an assessment tool and specificity of the feedback [9]. Reviewers identified specific training needs. In a critical analysis of a mini peer assessment tool in a clinical setting Abdullah pointed out that without adequate training feedback is entirely dependent on the skill of the person giving feedback his personal interest and previous experience [20]. Therefore training in skills of giving feedback is essential for proper implementation.

While studies in western settings have shown that acceptance of feedback depends on the credibility of the provider [9, 21] in a hierarchical culture such as that in Asia where the power distance between individuals at higher and lower social levels is greater it was not surprising to see a marked effect of hierarchy on acceptance of feedback [22]. Asian culture emphasises collectivity, maintaining the status quo and restraining actions that might disrupt the traditional order [23]. In an Indonesian replication of a Dutch feedback study it was found that in the Dutch study, it did not matter whether students received feedback from residents or specialists while in the Indonesian context feedback from specialists was rated more positively than feedback from residents [24, 25]. In this study hierarchical thinking was seen in both junior and senior examiners’ acceptance of feedback and reviewer delivery of feedback. Feedback given by senior reviewers was more likely to be valued. However a significant finding was that with more exposure to receiving constructive feedback the level of acceptance increased. It was also encouraging to see that some examiners were keen to overcome the cultural barriers such as hierarchical bias in order to carry out their professional duties competently.

Dissonance between qualitative and quantitative findings could be seen in two areas: regarding recruitment of reviewers and perceptions of hierarchy. This is perhaps explained by an overall acceptance of the peer feedback process by examiners as a challenging but necessary aspect of quality assurance. Examiners were willing to accept the process however they emphasised that careful attention should be given to reviewer selection and training, reviewer accountability, transparency and the ability to be equal partners within the process.

### Strengths and limitations of the study

Examiners were actively involved in the QA process at the time of gathering data reducing recall bias.

There were several limitations. The response rate for the questionnaire was 77%. This was mostly due to logistic difficulties in gathering examiners together for FGDs during an ongoing OSCE examination; however it is possible that some non responders may have had different views than those reported in the study.

Each year there are a limited number of examiners attending the exam in whichever country the exam is held. During the two examinations where data were collected for this study there were 23 and 26 examiners attending which is the reason for the limited number of participants from the various countries from whom data could be gathered.

One significant limitation of the study was that it was conducted during a period in which there was a lull in participation from Indian resource persons.

The focus group discussions were facilitated by the principal investigator DP who was a junior examiner on the OSCE exam. This may have introduced some bias. However this bias was offset to some extent by MA being the clinical exam convenor for the exam an examiner and peer reviewer while VW was the international development advisor for the exam. Findings were validated by discussion among all three investigators at different stages of the study.

## Conclusion

In our study examiners demonstrated a high level of appreciation of the peer feedback process although it was perceived as a stressful experience. In this setting cultural influences such as the concept of hierarchy had a significant impact on the acceptance of feedback. The process could be further refined to improve acceptability through scrupulous attention to training and selection of those giving feedback to improve the perceived validity of the feedback as well as to train reviewers in skills of giving feedback to enable better interpersonal dynamics and a more equitable feedback process. It is important to involve examiners in the training process to impart a better understanding of the importance of quality assurance and peer feedback. There was definite evidence that increasing exposure to receiving feedback led to better levels of acceptance.

The findings of this study led to increased awareness regarding the difficulties that both reviewers and examiners faced in negotiating the peer feedback process. The peer feedback process was restructured through the development of structured feedback forms, more rigorous recording of feedback and increased transparency and discussion between reviewers and examiners.

### Implications for future research and practice

The findings of this study highlight the need for careful consideration to be given to the context, selection and training of reviewers and the involvement of examiners as important stakeholders in the overall process in devising a peer feedback programme.

Further research to explore the utility, adaptation and acceptance of peer feedback programmes in medical education settings in South Asia could be useful.

## Additional file

Additional file 1: Peer feedback for examiner quality assurance on MRCGP[INT] South Asia - Questionnaire for examiners. (XLS 56 kb)

## Abbreviations

BD: Bangladesh
Examiner experience: 1: once, 2: twice, 3: three times, >3: more than 3 times
F: Female
FGDE: Examiner focus group discussion
FGDR: Reviewer focus group discussion
M: Male
MRCGP [INT] SA: South Asian International membership examination of the Royal College of General Practitioners of United Kingdom
OSCE: Objective structured clinical examination
Pak: Pakistan
SL: Sri Lanka
UK: United Kingdom
